# A new manual method for assessing elbow valgus laxity

**DOI:** 10.1186/1758-2555-4-11

**Published:** 2012-03-19

**Authors:** Kenji Yasui, Teruhisa Mihata, Atsushi Takeda, Chisato Watanabe, Mitsuo Kinoshita

**Affiliations:** 1 Department of Orthopedic Surgery, Osaka Medical College, Takatsuki, Osaka, Japan; 2 Department of Rehabilitation Medicine, Veritas Hospital, Kawanishi, Hyogo, Japan

## Abstract

**Background:**

A screening of ulnar collateral ligament insufficiency is required for overhead throwers, since secondary pathologic changes result from an increased elbow valgus laxity. We developed a new manual method for assessing elbow valgus laxity and investigated the reliability of this method and its correlation with ultrasonographic assessment.

**Methods:**

We defined elbow valgus laxity as the difference between the shoulder external rotation angle (ER angle) measured with the elbow in 90 degrees flexion and that measured with the elbow in extension because ER angle measured with the elbow in 90 degrees flexion includes elbow valgus laxity and ER angle with the elbow in extension does not include it. ER angle measurement with the elbow in extension involved the use of a custom arm holder. Three examiners each measured elbow valgus laxity by the new method in 5 healthy volunteers. Intraobserver and interobserver reliability was evaluated by calculating the intraclass correlation coefficient. We then assessed 19 high-school baseball players with no complaints of shoulder or elbow pain. Elbow ultrasonography was performed with a 10-MHz linear transducer with the elbow in 90 degrees flexion, and the forearm in the neutral position, and the width of the medial joint space at the level of the anterior bundle was measured. Elbow valgus laxity assessed by ultrasonography was defined as the difference between the medial joint space width with gravity stress and that without gravity stress. Increased elbow valgus laxity assessed by both our method and ultrasonography was defined as the difference between the laxity of the elbow on the throwing side and that on the contralateral side. Pearson's correlation coefficient (r) was calculated to evaluate the relationship between increased elbow valgus laxity obtained by our manual method and that by ultrasonography.

**Results:**

Intraobserver reliability ranged from 0.92 to 0.98, and interobserver reliability was 0.70. The increased elbow valgus laxity assessed by our method was significantly correlated with that assessed by ultrasonographic assessment (*P *= 0.019, *r *= 0.53).

**Conclusions:**

Elbow valgus laxity can be assessed by our method. This method may be useful for screening for insufficiency of the ulnar collateral ligament.

## Background

Overhead-throwing athletes risk ulnar collateral ligament (UCL) injury due to tremendous elbow valgus stresses during the late cocking and acceleration phases of the throwing motion. These valgus forces at the elbow have been estimated to be as high as 64 to 120 N m [1-3]. Repetitive stresses can cause attenuation or tearing of the UCL, resulting in UCL insufficiency [4-6]. This condition causes elbow pain during throwing, as well as other secondary changes, including ulnar nerve symptoms, medial epicondylitis, olecranon osteophytes, osteochondritis dissecans of the capitellum, and loose bodies [7-10]. Therefore, accurate evaluation of elbow valgus laxity is important for diagnosing UCL insufficiency. Imaging studies, such as stress radiographs and ultrasonograms, have been used to evaluate elbow valgus laxity [11-16], and diagnostic methods such as arthrography, MRI, and CT arthrography are used to diagnose UCL injury [17-19]. However, evaluating elbow valgus laxity solely by using existing physical methods, such as the elbow valgus stress test, is challenging, because adequately stabilizing the humeral rotation at the required elbow flexion angles is difficult [1,20].

When the shoulder is external rotated with the elbow in 90 degrees flexion, valgus stress is imparted to the medial elbow. The UCL restrains valgus stress from 30 to 120 degrees flexion, and valgus instability is most apparent from 70 to 90 degrees flexion [1,2], therefore, increased elbow valgus laxity from UCL insufficiency affects the shoulder external rotation angle (ER angle) with the elbow in 90 degrees flexion. In our previous cadaveric study, transection of the UCL increased the measured ER angle with the elbow in 90 degrees flexion, although the glenohumeral joint condition was not changed [21]. In contrast, measurement of the ER angle with the elbow in extension includes less elbow valgus laxity, because bony articulation provides stability, particularly during extension [22,23]. For the reasons stated above, we surmised that elbow valgus laxity can be assessed by comparing the shoulder ER angle when the elbow is at 90 degrees flexion with that when the elbow is in extension. To confirm whether our method is reliable, we aimed to (1) investigate the intra- and interobserver reliability of the assessment of elbow valgus laxity by our method and (2) evaluate the correlation between the elbow valgus laxity determined by our method and that by ultrasonography which can accurately assess elbow valgus laxity by measuring the medial joint space of the elbow during gravity [14] or valgus stress [12,24].

## Methods

### New method for assessing elbow valgus laxity

**Figure 1:**
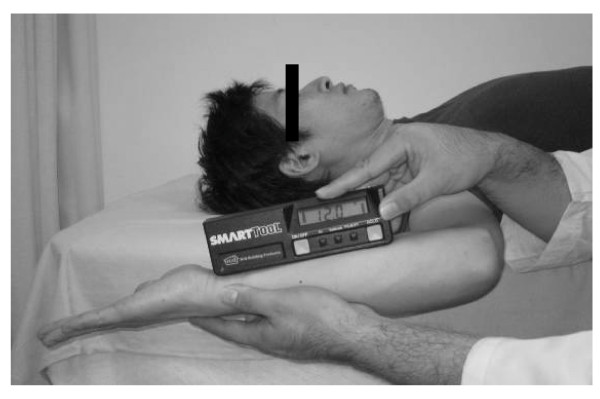
**Use of a digital inclinometer to measure ER angle during flexion**. Subjects were in the supine position with the arm abducted 90 degrees and the forearm in the neutral position.

Our method compares the ER angle when the elbow is at 90 degrees flexion with that when the elbow is in extension. The ER angle was measured with the elbow in 90 degrees of flexion and in extension in the supine position with the arm abducted 90 degrees. The ER angle during flexion was measured with the forearm in the neutral position by using a digital inclinometer (Smart Tool, M-D Building Products, Oklahoma City, OK; accuracy, 0.1 degrees). The distal edge of the digital inclinometer was placed at the wrist crease, with the instrument along the midline of the forearm (Figure 1).

**Figure 2:**
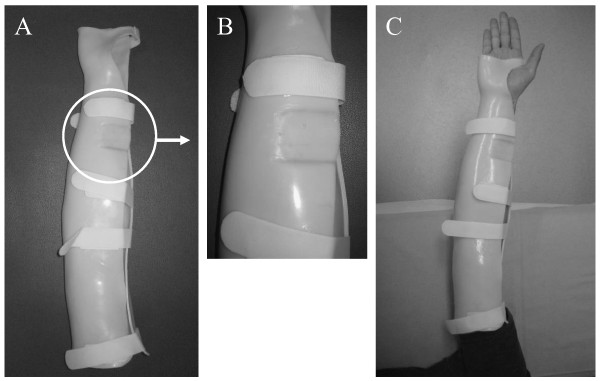
**A custom-designed arm holder made of thermoplastic resin (A)**. A groove on the arm holder made it possible to measure the rotation angles accurately **(B)**. This arm holder can maintain the subject's elbow in extension with the wrist in the neutral position **(C)**.

**Figure 3:**
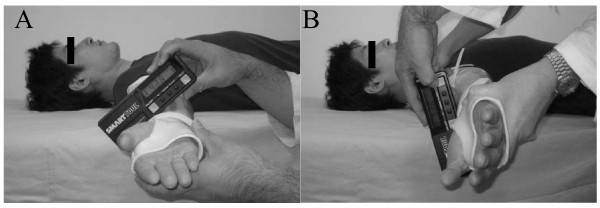
**Use of a digital inclinometer to measure ER angle during extension**. The ER angle during extension was the difference in the inclination of the arm holder at an extension-method ER angle of 0 degrees **(A) **and that of at the end range **(B)**. A groove in the forearm region of the arm holder enabled reproducible positioning of the inclinometer and thus increased the reliability of data.

To hold the arm in the extended position and to restrict the forearm motion, we used a custom-designed arm holder made of thermoplastic resin that was capable of maintaining the subject's elbow in extension with the wrist in the neutral position (Figure 2A, C). This arm holder was applied after the subject's arm had been abducted 90 degrees with the elbow extended. Zero degrees of ER angle with the elbow in extension was defined as that when the arm position placed the hand parallel to the floor (Figure 3A). The maximum ER angle during extension was calculated as the difference between the inclination of the arm holder at the end range and that at an ER angle of 0 degrees (Figure 3B). The inclinometer was placed at a groove on the arm holder to measure the rotation angles accurately (Figure 2B). By the use of this arm holder, we could measure shoulder ER angle with the elbow extended position excluding forearm rotation.

ER angle was measured with a posteriorly directed force on the anterior aspect of the shoulder to stabilize the scapula consistently by one examiner and another examiner placed the digital inclinometer. The end range of the ER angle was defined as the point of cessation of rotation or when scapular movement could be appreciated. We defined elbow valgus laxity as the difference between the ER angle during flexion and that during extension, because the ER angle measured under flexion includes any elbow valgus laxity [21].

### Intra- and interobserver reliability of our new method for assessing elbow valgus laxity

To determine the reliability of our method, 3 physical therapists well trained in measuring the ER angle with the elbow in extension assessed the elbow valgus laxity in 5 volunteers (2 men and 3 women) using our method. Informed consent was obtained from all subjects. The mean age of the subjects was 33.6 years (range, 26 to 46 years), and they had no history of shoulder or elbow pain or trauma. Four subjects were right-handed, and 1 was left-handed. The 3 examiners each measured the ER angles with the elbow at 90 degrees flexion and in extension bilaterally and calculated the accompanying elbow valgus laxity 3 times at 1-week intervals. The intra- and interobserver reliabilities of our assessment method were evaluated by using the intraclass correlation coefficient (SPSS version 13.0, SPSS, Chicago, IL).

### Correlation with ultrasonographic assessment

To confirm that the difference between the ER angles during flexion and extension does in fact represent elbow valgus laxity, the correlation between elbow valgus laxity as assessed by our manual method and that by ultrasonography was evaluated. Institutional Review Board approval was obtained before we began the investigation, and written informed consent was obtained from subjects and their parents.

Nineteen male high-school baseball players with no complaints of shoulder or elbow pain and no history of injury volunteered for the study. The average age at the time of study was 16.4 years (range, 15 to 17 years). The average duration of their active participation in baseball was 8.2 years (range, 6 to 10 years). Sixteen subjects were right-handed; the remaining 3 were left-handed. Four subjects were pitchers, and the remaining 15 were fielders; none of the subjects had general joint laxity, according to the system of Carter and Wilkinson [25].

**Figure 4:**
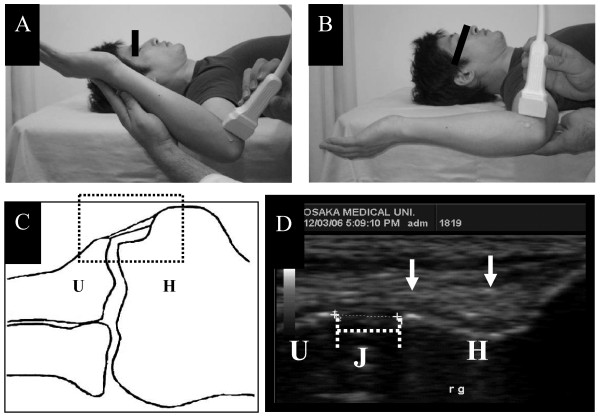
**Ultrasonography of the elbow (A) without gravity stress (shoulder at 60° of external rotation) and (B) under gravity stress**. **(C) **Sketch and **(D) **ultrasonogram (region encompassed by dotted box in C) of the medial elbow. Arrow, Anterior bundle of the ulnar collateral ligament; H, humerus; U, ulna; J, joint space (dotted line).

Elbow ultrasonography was performed as described previously [14] with a 10-MHz linear transducer (LOGIQ Book, GE Healthcare, Waukesha, WI) by a single shoulder and elbow surgeon (C.W) who was well skilled in the technique and blinded to the subject's throwing side. The subjects were placed supine on the exam table, with the shoulder in 90 degrees abduction, the elbow in 90 degrees flexion, and the forearm in the neutral position. The transducer was placed on the medial aspect of the elbow and a long-axis scan of the anterior bundle of the UCL was obtained. The width of the medial joint space at the level of the anterior bundle was measured (Figure 4).

To accurately determine elbow valgus laxity, the subjects were relaxed and measurements were performed both with and without gravity stress. Without gravity stress, the examiner passively held the subject's arm at 60 degrees of shoulder external rotation. None of the subjects experienced elbow pain during the examination. Ultrasonographic measurements were made with electronic calipers. Elbow valgus laxity assessed by ultrasonography was defined as the difference between the medial joint space distance with gravity stress and that without gravity stress.

A single well-trained physical therapist (A.T) who was blinded to the subject's throwing side then used our new assessment method to measure the ER angles during flexion and extension in all subjects. Measurements were obtained 3 times, and the mean value was used in calculations of elbow valgus laxity. Both ultrasonographic assessment and manual assessment were blinded assessment regarding the throwing side.

Increased valgus laxity was calculated by comparing between dominant side and non-dominant side. Pearson's correlation coefficient (r) was calculated to evaluate the relationship between increased elbow valgus laxity obtained by our manual method and that by ultrasonography (STATISTICA version 6.0; StatSoft Inc., Tulsa, OK). A *P *value of less than 0.05 was considered to indicate statistical significance.

## Results

### Intra- and interobserver reliability of new manual method for assessing elbow valgus laxity

The intraobserver reliabilities (based on the interclass correlation coefficient) for each of the 3 examiners that used our manual method for assessment of elbow valgus laxity were 0.92, 0.95, and 0.98. The interobserver reliability was 0.70.

### Correlation with ultrasonographic assessment

**Figure 5:**
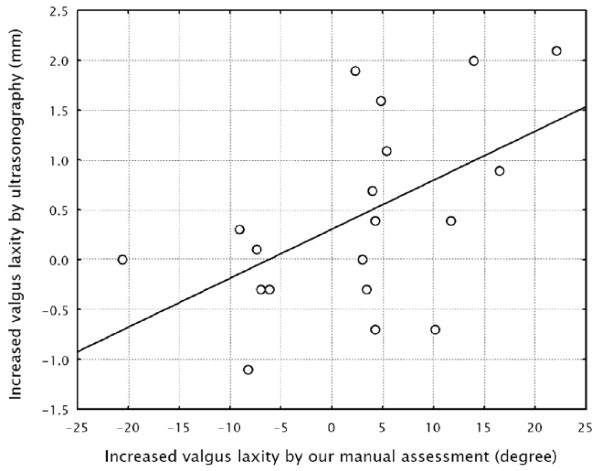
**Increased elbow valgus laxity was defined as the difference between the laxity of the elbow on the throwing side and that on the contralateral side**. Increased elbow valgus laxity as measured by our method was significantly correlated with that assessed by ultrasonography in the high-school baseball players (*P *= 0.019, *r *= 0.53).

Among the 19 male high-school baseball athletes assessed, the increased elbow valgus laxity assessed by ultrasonography on the throwing side was 0.4 ± 0.2 mm(mean ± SE) and the increased elbow valgus laxity assessed by our method on the throwing side was 2.4 ± 2.4 degrees. The increased elbow valgus laxity assessed by our method was significantly correlated with that assessed by ultrasonographic assessment (*P *= 0.019, *r *= 0.53; Figure 5).

## Discussion

Most elbow injuries in throwing athletes are overuse injuries [15,26,27]. Repetitive valgus stress can cause attenuation, tearing, or insufficiency of the UCL, as seen in throwing athletes. Therefore, evaluation of elbow valgus laxity is important during medical examinations of both symptomatic and asymptomatic throwing athletes to prevent overuse injuries in the elbow joint. However, current physical examination methods, such as the elbow valgus stress test, are poor tools for diagnosing increased elbow valgus laxity [1,20], especially asymptomatic elbows; stress radiography or ultrasonography have therefore been used for this purpose [11-16].

Stress radiography of 40 uninjured professional baseball pitchers revealed that the difference in the medial joint space opening between stressed and unstressed elbows on the throwing side was significantly greater than that on the contralateral side (0.32 ± 0.42 mm) [11]. However, the authors reported that this small difference likely would be almost unidentifiable if manual orthopedic laxity tests alone had been used.

Ultrasonographic assessment of the UCL in college baseball players showed that the medial joint space was significantly wider on the throwing side (2.7 mm) than on the contralateral side (1.6 mm; *P *< 0.01) [14]. Similarly, ultrasonographic assessment of the elbow under valgus stress revealed widening of the medial joint space on the throwing side (4.2 mm) compared with the contralateral side (3.0 mm; *P *< 0.01) in asymptomatic major-league baseball pitchers [12]. As these previous studies show, ultrasonography can be used to quantitatively assess elbow valgus laxity by measuring the width of the medial joint space, and even asymptomatic baseball players have some acquired laxity.

Our present study showed the high level of intra- and interobserver reliability of our new manual assessment method. In addition, in the high-school baseball players that we evaluated, the difference between ER angle with the elbow in 90 degrees of flexion and that with the elbow in extension was correlated with the widening of the medial joint space measured by ultrasonography. These results suggested that elbow valgus laxity can be assessed manually and reliably by our new method.

Various physical examination methods are used to diagnose UCL insufficiency, including the recently developed "milking maneuver" [2,28] and "moving valgus stress test" methods [6]. Although perhaps useful for diagnosing symptomatic UCL insufficiency, these physical assessment methods are pain reproducing tests and cannot be used to diagnose asymptomatic UCL or measure the degree of laxity. Even asymptomatic UCL insufficiency may cause secondary pathologic change. In contrast, our manual assessment method may be useful for detecting increased elbow valgus laxity during medical examinations of asymptomatic throwers because our method is not a pain reproducing test and can measure the degree of laxity quantitatively.

A limitation of our method is that it requires a custom arm holder and digital inclinometer for measuring the ER angle with the elbow in extension. We currently are evaluating modifications to this method in order to accomplish manual assessment of elbow valgus laxity without the need for a customized arm holder. Another limitation is the subjects of this study were asymptomatic high school baseball players, therefore, with the validity of the results in symptomatic sportsmen needs to be determined. However, as reported by Sasaki et al. [14], the width of the medial joint space is generally increased to a certain degree in symptomatic baseball players, as compared to asymptomatic baseball players; we believe that assessment of elbow valgus laxity in symptomatic baseball players by our method will be higher, as compared to asymptomatic baseball players. Another limitation of our study is that the subjects in the reliability assessment study were considerably older than those in the ultrasonographic assessment study (mean ages, 33.6 and 16.4 years, respectively). Because shoulder range of motion decreases with age [29], the reliability of our method in younger subjects may differ from that presented here.

## Conclusions

The results of assessment of elbow valgus laxity in baseball players by our new manual method were correlated with ultrasonographic data. Our new method likely will be useful for screening of asymptomatic UCL insufficiency, especially in throwing athletes.

## Competing interests

The authors declare that they have no competing interests.

## Authors' contributions

KY participated in data collection, and drafted the manuscript. TM conceived the main idea, participated in data collection, and in the revision of the manuscript. AT and CW participated in data collection. MK participated in the revision of the manuscript. All authors read and approved the final manuscript.
